# Aging, senescence, and cutaneous wound healing—a complex relationship

**DOI:** 10.3389/fimmu.2024.1429716

**Published:** 2024-10-17

**Authors:** Steven O’Reilly, Ewa Markiewicz, Olusola C. Idowu

**Affiliations:** Hexislab Limited, The Catalyst, Newcastle Upon Tyne, United Kingdom

**Keywords:** skin wound, wound healing, senescence, SASP (senescence-associated secretory phenotype), inflammation

## Abstract

Cutaneous wound healing is a complex multi-step process that is highly controlled, ensuring efficient repair to damaged tissue and restoring tissue architecture. Multiple cell types play a critical role in wound healing, and perturbations in this can lead to non-healing wounds or scarring and fibrosis. Thus, the process is tightly regulated and controlled. Cellular senescence is defined as irreversible cell cycle arrest and is associated with various phenotypic changes and metabolic alterations and coupled to a secretory program. Its role in wound healing, at least in the acute setting, appears to help promote appropriate mechanisms leading to the complete restoration of tissue architecture. Opposing this is the role of senescence in chronic wounds where it can lead to either chronic non-healing wounds or fibrosis. Given the two opposing outcomes of wound healing in either acute or chronic settings, this has led to disparate views on the role of senescence in wound healing. This review aims to consolidate knowledge on the role of senescence and aging in wound healing, examining the nuances of the roles in the acute or chronic settings, and attempts to evaluate the modulation of this to promote efficient wound healing.

## Introduction

With chronological aging, changes in the subcellular structures and functions of cells occur. Cellular senescence, a key hallmark of aging, is now appreciated to play a critical role in many physiological processes, age-related diseases, and regeneration. Senescence is defined as reversible cell cycle arrest and phenotypic changes, epigenetic changes, and the senescent-associated secretory phenotype (SASP). Senescence is there to retard oncogenesis defined by proliferative arrest. Initially described by Hayflick and Moorhead after serial passaging of cells *in vitro* (1, 2), they thought it was an artifact of cell culture but appreciated that this was not the case. Presently, senescence as a scientific field is burgeoning with thousands of scientific publications in the recent years alone. Remarkable progress in the last few years in senescence and its regulation in disease and homeostasis has occurred.

It is now appreciated that senescence is necessary for normal development (3–5), but it is also associated with a variety of age-related diseases such as dementia and idiopathic pulmonary fibrosis (6, 7). Senescence has also been shown to play a key role in not only cutaneous wound healing (8) but also fibrosis (9–11). These two phenomena are at the opposite ends of the spectrum, and this may relate to either acute or chronic senescent cells at the site of wounding. Although senescence is viewed as a negative event in terms of age-related diseases (12–15), it appears to have positive benefits in terms of promoting cutaneous wound healing, i.e., in acute wound healing, senescence promotes healing. This review aims to analyze the role of cellular senescence and aging in wound healing, emphasizing its duality depending on the specific context.

## Senescence characteristics

Senescence is an irreversible state of cell cycle arrest, even in the presence of growth factors, that provides a barrier to tumor formation. It is associated with a variety of phenotypic changes including apoptosis resistance, telomere attrition, altered metabolism (16), perturbed mitochondria, enhanced glycolysis, elevated reactive oxygen species (17), and SASP (12, 14, 18). The SASP is a complex blend of inflammatory cytokines and lipids that are synthesized and released from senescent cells (19). The exact composition of the SASP is varied and can differ depending on the cell type and the senescent induction agent used, and although a precise definition has not been achieved, it is generally accepted to contain IL-6, IL-1β, IL-8, TGF-β, matrix metalloproteases (MMPs) bioactive lipids (e.g., ceramides and prostaglandins), serpines, and microRNAs that contribute to inflammation (19–22). The SASP is thought to be a key hallmark of senescent cells, but its regulation is not well defined and is context dependent, but in many cases it is reinforced by NF-KB signaling (15). Different upstream senescence drivers may result in different compositions of the SASP, and most importantly, the SASP can act on non-senescent cells in a paracrine fashion. In this way, senescent cells can transmit signals to other non-senescent cells to become senescent (23).

Metabolic alterations in senescent cells include enhanced glycolysis. It has been determined that there are changes in autophagy, although anti- and pro-senescent effects of autophagy have been reported (24) and likely reflect the complexity of senescence and cell specificities. Furthermore, in senescent cells, heterochromatin foci form (25). Telomere attrition is also a significant hallmark of senescent cells, and introduction of the enzyme telomerase into cells retarded senescence and increased the lifespan of these cells (26). Another classic hallmark of senescence is impaired nuclear integrity and formation of heterochromatin foci. Unfortunately, no one specific marker is diagnostic for senescent cells, and because of the heterogeneity in the composition of the SASP, this also is not a complete marker. Rather, an approach based on multiple different markers combined, such as P16, P21, DNA damage, γH2AX, elevated ROS, and metabolic alterations such as glycolysis, is often used. Although p16 is often used as a marker for senescence and is very validated (27), it is important to note that p16 is not solely exclusive to senescent cells as this also plays a role in cell cycle control and can be independent of senescence (28). Unprecedented levels of research are currently ongoing to discover a specific and wholly reliable marker of senescence.

## Wound healing

The skin is the largest organ of the body with a surface area of approximately 1.5–2 m^2^, forming a physical barrier between the host and the external environment. It is the primary interface between the host and the environment and protects from chemical insult, microbial insult, and ultraviolet radiation (UVR) as well as provides thermoregulation. Wound healing is a normal physiological response that follows in coordinated phases. Although complex and intricate, it is divided into four distinct but overlapping phases—(1) hemostasis, (2) inflammation, (3) proliferation, and (4) remodeling—which ultimately result in the healing of the wound and regaining of structural integrity (29). Hemostasis is the primary event after the initial trauma. The second phase of wound healing is inflammation. This inflammation occurs 1 to 3 days after the initial wound. Neutrophils are the first primary and most abundant immune cells recruited into the wound (30); they are attracted to the wound by the release of DAMPs [such as high-mobility-box-1 (HMGB-1), uric acid, etc.].

The third phase is the proliferation phase. In this phase, formation of granulation tissue occurs with collagen and other ECM protein deposition primarily mediated by a specialized type of fibroblast that has transitioned from a quiescent to an activated state: myofibroblast. Finally, remodeling occurs; this is where the granulation tissue is replaced by the scar tissue.

## Acute transient senescence drives a homeostatic regenerative response

Although senescence is primarily associated with aged-related diseases, it has been found to be important in promoting wound healing. One of the first descriptions of the role of senescence in wound healing was published in 2014. Demaria et al. created a mouse model in which senescent cells can be identified *in vivo* and eliminated *in vivo*: the p163MR mice (31). p16^INK4^-positive cells can be essentially identified and killed *in vivo* by ganciclovir, which results in the death of these cells. When the authors performed full-thickness punch biopsies on the dorsal flanks of mice, they found that senescence cell markers were very high at 3 days post-wound, peaked around day 6, and returned to barely detectable levels by day 9 through 12 (31). This correlated with the mRNA expression of p16 and p21 levels. The selective elimination of these senescent cells retarded wound healing. Furthermore, using a double p16 and p21 knockout mouse, they also showed significant retardation of wound healing (31). The p16 and p21 double knockout mouse has barely detectable senescent cells and either alone knocked out can compensate for the other, and the single p16 or p21 KO mouse did not retard wound healing (32). Finally, the authors identified that the positive senescent cells in the wound are primarily fibroblasts and endothelial cells that are the primary senescent cells. They further identified that the senescent fibroblasts secrete high levels of PDGF-A as a critical SASP-contained factor (31). This senescent-derived PDGF-A drives the activation of myofibroblasts from fibroblasts as topical wound application of PDGF-A to healing wounds in the mice eliminated for senescent cells increased the number of myofibroblasts and restored the wound healing kinetics to that of control. This work suggests that early induction of senescent cells is important especially in the proliferation phase (phase 3) as removal of senescent cells impaired wound healing (31). However, it has been shown in mice that senescence cells are induced in wound healing to curb fibrosis through the matricellular protein CCN1 (8). CCN1 knock in mice that express a senescence-defective CCN1 mutant had less senescent cells and fibrosis compared to wild-type mice (8). CCN1 appears to reduce fibrosis via triggering senescence in hepatic cells through the engagement of specific integrins (33). This phenomenon of senescence cells in healing and then acting to retard fibrosis has been found in the liver also (34). Moreover, it was found that SASP from senescent cell transferred to keratinocytes promoted regeneration and stem-like features; however, prolonged stimulation over a chronic period of time was detrimental (35), indicating that acute versus chronic tissue senescence mediates differential effects. This is further compounded in age, where in the skin there is a general increase in senescent cells with increasing age (36). Interestingly, it was found recently that thrombospondin-1 was identified as a key regulator of dermal fibroblast senescence via the activation of the SMAD4 transcription factor (37).

## Chronic senescence drives altered wound healing

**Figure 1:**
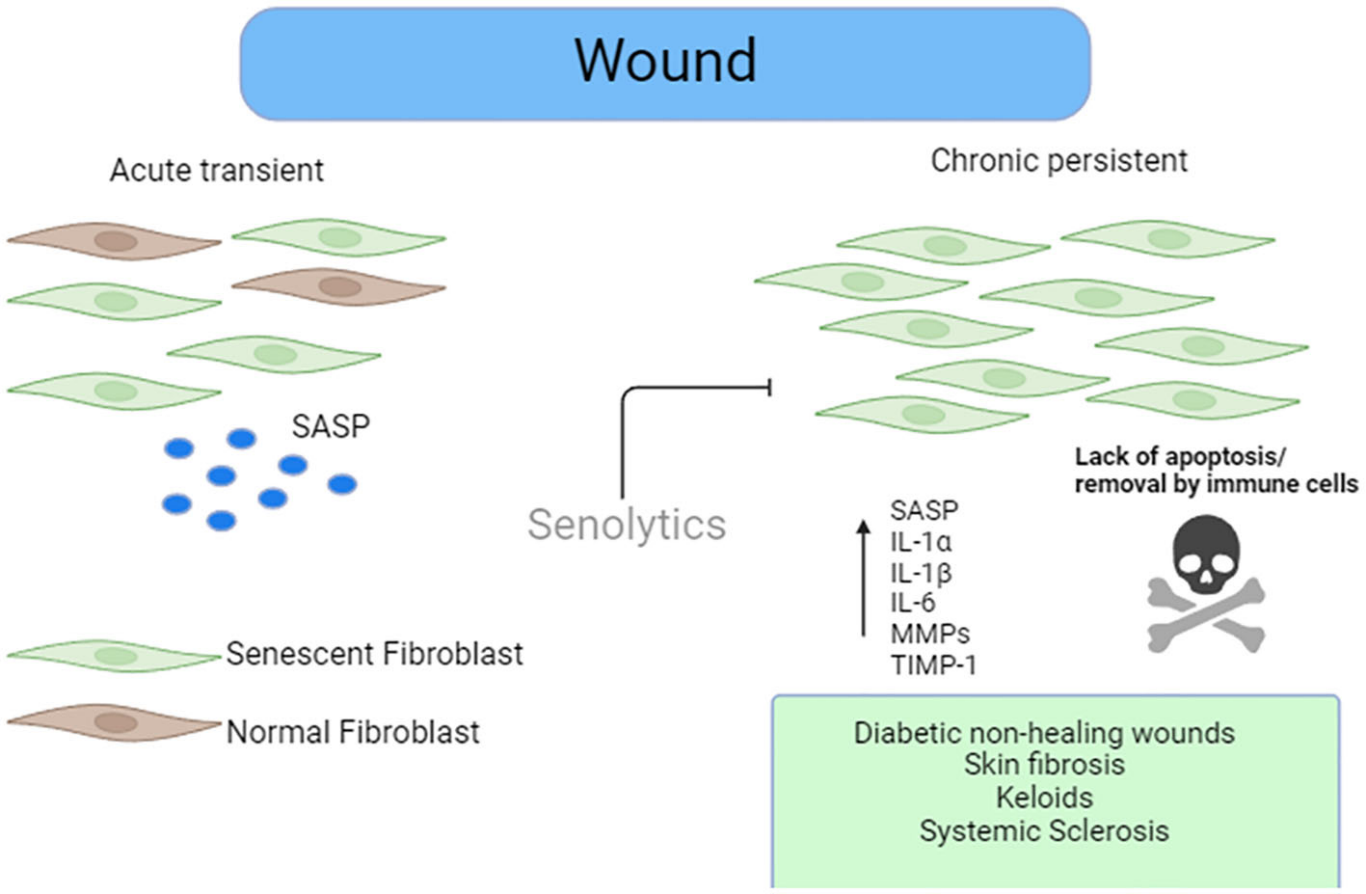
Role of senescence in wound healing. Senescent stromal cells are present in acute wound healing that facilitate efficient wound repair via factors released in the SASP. Removal of these cells in the acute stage of wound repair retards efficient wound healing. However, the persistence of the senescent cells in the later stages of the wound healing stages leads to either chronic non-healing wounds such as what is seen in diabetics or exuberant ECM deposition leading to fibrosis such as that seen in SSc or keloids. Senolytics could eliminate these cells, leading to reduced burden and improved wound healing.

It is clear in the acute setting that senescence is necessary for the appropriate wound healing response through secretion of SASP factors which include PDGF-A (31). However, lingering senescent cells at the site of the wound may impede wound healing or lead to fibrosis (**Figure 1**). The effect in the skin of reduced senescence via CCN1 knock in mice revealed that senescence appeared to be a mechanism to control unabated wound healing and fibrosis (8). In other models of healing, such as the liver paracrine, senescence driven by SASP-derived TGF-β inhibited wound healing (38). The differences here between senescent cells may be due to acute and chronic senescent cells and their clearance after an acute wound through immune clearance of senescent cells (39).

The delayed healing of chronic wounds is common among diabetics and the elderly (40). This is associated with significant morbidity. Chronic wounds are defined as those that do not progress through the normal phases of healing and therefore fail to heal in a timely manner. Furthermore, the associated healthcare costs are significant, with many diabetics suffering from chronic non-healing wounds (41). It was demonstrated in the diabetic delayed wound healing mouse model that, compared to non-diabetic mice, there were elevated senescent cells in the wound coincident with delayed wound healing. Furthermore, a great proportion of senescent cells were macrophages, and these were showing an enhanced SASP secretome (42). Furthermore, SASP was enriched for CXCL1 and CXCL2 along with the receptor CXCR2, and conditioned media from these senescent cells transferred to dermal fibroblast led to an elevated ECM gene expression (42). Finally, in the *in vivo* delayed wound healing mouse model, pharmacological blockade of CXCR2 with SB265610 enhanced the delayed wound healing and reduced senescence and inflammation (42). The chronic inflammatory response that is in the diabetic chronic wound helps facilitate a chronic senescent cell population (43). The direct induction of senescence and SASP was indeed demonstrated in endothelial cells by hyperglycemia that may be mediated by enhanced ROS (44). Remarkably, transfer of senescent irradiated fibroblasts to young mice in the skin leads to increased senescence and a significantly delayed healing compared to non-senescent transplanted cells (45), and even after healing there were chronic senescent cells. Diabetic non-healing wounds have been found to harbor senescent cells (46), and a recent study demonstrated a clear upregulation of senescent cell markers in diabetic non-healing wounds with disorganized fibroblasts. Furthermore, the authors demonstrated that senescent cells within the non-healing wound showed reduced ferroptosis and impaired ferritinophagy (47). Ferroptosis is a specific form of iron-dependent cell death driven by alterations in iron handling and redox homeostasis (48). Thus, the repression of senescence cell ferroptosis would promote a lingering sustained senescent cell environment that would retard efficient wound healing. Underpinning the retarded ferroptosis is impaired ferritinophagy—a lysosomal breakdown of ferritin—and this has been found to be displayed in senescent cells before where the senescent cells are highly resistant to cell death (49). Elevated senescent cells have also been described in non-healing venous ulcers—a major clinical problem (50). Mining a publicly available database of whole-RNA sequencing data derived from wound edge of diabetics with foot ulcers involved versus diabetics with non-involved skin biopsies, the authors found that senescence gene networks were significantly elevated compared to cases with non-involved skin biopsies (51). Recently, a study identified a key role for senescence in diabetic wound healing with senescent adipocytes playing a key role. Transplantation of adipose tissue from diabetic mice to non-diabetic mice impaired wound healing and was associated with a significant increase in SASP factors (52), suggesting that adipocyte senescence is important in cutaneous wound healing. Non-diabetic chronic wounds include pressure ulcers, radiation ulcers, and venous ulcers. Wang et al. demonstrated increased senescent cells in radiation ulcers and that the nucleoside analogue cordycepin reduced ulcers by preventing cell senescence via increased nrf-2 expression—a key transcription factor (53). Furthermore, in an animal model of radiation-induced ulcers, senolytic therapy mitigated fibrosis (54). It appears that, in radiation injury, senescent cells promote dysregulated healing via cytokine IL-33 (55). Also, in venous pressure ulcers, senescent cells are present within the tissue in significant numbers, indicating that they could play a pathological role (56).

In experimental models of kidney fibrosis, it was found that senescent cells are elevated in the kidney tissue, and clearance of these senescent cells with senolytic therapy led to reduced kidney fibrosis (57, 58). Senescence has long been associated with pulmonary fibrosis which can be seen as a maladaptive wound response (9, 10). Indeed the selective elimination of senescent cells via the senolytic cocktail dasatanib and quercetin in mouse models of lung fibrosis led to significantly reduced senescent cells and fibrosis, underpinning the importance of senescence (59).

In the autoimmune disease systemic sclerosis (SSc), there is a maladaptive fibrosis occurring in the skin that leads to chronic skin fibrosis. Elevated senescent cells have been demonstrated in the skin of these patients (11, 60). The number of senescent cells correlated with the fibrosis score also (11). It was also demonstrated that enhanced ROS and oxidative stress mediates the enhanced senescence found in skin fibrosis (61). Furthermore, it was found that *in vitro* culture of senescent SSc dermal fibroblasts with senolytics reduced ECM deposition, suggesting a clear functional role of senescence in mediating skin fibrosis (62). Only one study in cutaneous fibrosis using senolytics has been undertaken. This was using the bleomycin model of skin fibrosis and navitoclax. Here the authors demonstrated that navitoclax led to reduced cutaneous fibrosis in this animal model, consistent with the reduction of the myofibroblast cell type (63)—the cell type primarily responsible for ECM production. This would indicate that the removal of these cells—the myofibroblast—is beneficial; however, it was not appreciated at that time that navitoclax is a senescent cell removal agent, and thus the authors did not examine directly the senescent markers in the myofibroblasts (63). It is tempting to speculate in the cutaneous fibrosis model that the senescent myofibroblasts were the target of navitoclax and the majority of senescent cells are the myofibroblasts, although this assumption requires experimental evidence.

The different outputs of senescent cells appear to be related to chronicity, and in the case of non-healing wounds in diabetes, it has been shown that alteration in specific cell death pathways such as ferroptosis is retarded, which promotes senescent cell persistence (47). Impaired ferroptosis and the removal of senescence cells are related to perturbed ferritinophagy—lysosomal degradation of ferritin (47, 49) and iron perturbation are found in diabetic wounds (64). In the context of aged chronic non-healing wounds, questions regarding the induction of senescent cells remain, such as the following: (1) Is it that all cells in the wound are senesced by high glucose? (2) Is only a subset of cell types rendered senescent by high glucose? (3) Does removal of the hyper-glucose environment reverse the senescent cells? Currently, there is no clear answer to these questions, and this is an important area of research.

It is obvious that removing senescent cells in the acute phase of wound repair is detrimental to efficient repair, but removing these later on can help in skin fibrosis (63). This was shown in the bleomycin skin fibrosis model with the senolytic navitoclax; however, at this time, it was unappreciated that this drug was a senolytic, so linking the two concepts together was not evaluated (63). Further studies examining skin wound healing in the chronic state, either in the diabetic rodent models or in fibrosis with the intervention of senolytics, need to be performed to clarify the precise role of senescent cells. The sometimes-opposing roles of senescence on wound repair appear nuanced and time-dependent. Mechanistic studies on the role of the SASP also need clarification to determine the role of the SASP in mediating the effects of senescent cells, and if they are negative, this would give a rationale for the use of senomorphs—blocking the senescent cell released SASP. Few senomorphs have been described, but among this small list is the mTOR inhibitor, rapamycin (65), that selectively blocks the SASP. If indeed the SASP is playing a key role in perturbed wound healing, then targeting this specifically with senomorphs such as rapamycin would be beneficial. Furthermore, metformin has also been proposed as a senomorphic agent, and application of metformin accelerated wound healing in the diabetic db/db mouse model of impaired wound healing (66). This suggests that metformin could be a promising facile senotherapeutic. Vitamin D has also recently been demonstrated to retard in dermal fibroblasts the SASP release from these cells by blocking the p38 pathway (67). Much is still to be understood in relation to senescence and the SASP and their impact in chronic wounds and maladaptive repair. Pre-clinical studies with senolytics or senomorphs given at distinct times in the repair processes in skin healing must be employed to attribute their precise roles.

## Senescent cells in aging and photoaged skin

Although it appears that senescent cells in the initial phase of wound healing play a key role in efficient regeneration and lingering, senescent cells in the wound is deleterious. It has become clear that, simply with chronological aging, senescent cells accumulate in the skin with increasing age (68–70). Skin aging is associated with intrinsic factors and external stressors, such as UV exposure, that lead to progressive loss of skin integrity and a reduced regenerative capacity. UV rays make up the smallest fraction of the solar spectrum, but they are the most damaging. Aged skin has specific phenotypic features including thinning of the epidermis (71), loss of collagen content, and gain of elastic fibers—all of these can lead to the features of aging such as wrinkles and laxity with a “sagging” appearance. Extrinsic aging is provoked by factors such as UV exposure, smoking, and air pollution where they exert an aging effect.

**Figure 2:**
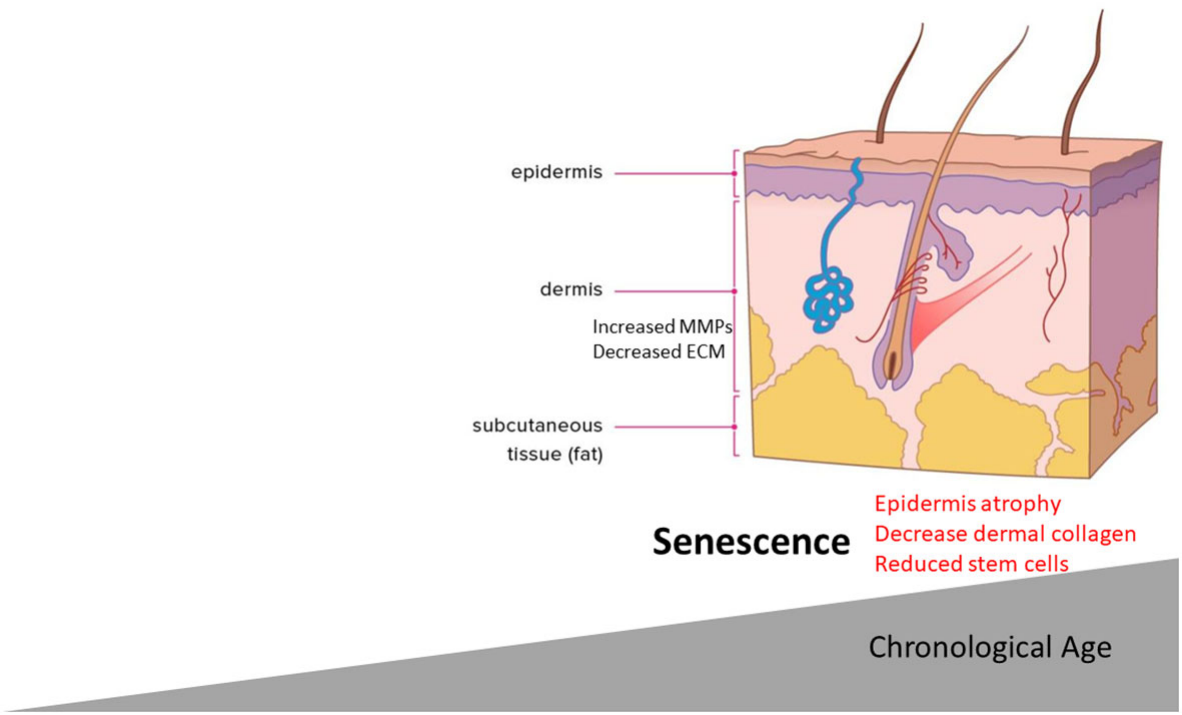
Aging skin primary features. The role of senescence could mediate the aged phenotype.

Given the presence of senescent cells in aged skin and the phenotypic features of aged skin, it seems plausible to link the two events (**Figure 2**). This, however, has been explored less so. It is known that there is a significant reduction of ECM and collagen fibrils in aged skin. Most recently, a key link between senescence and skin aging features such as epidermal thinning has been established. It was found that, in aged skin, melanocytes—responsible for melanin production—had higher levels of senescence associated with reduced telomeres (72).

Fascinatingly, senescent melanocytes could transmit senescence to other cells via the SASP and CXCR3-dependent mitochondrial ROS generation that led to proliferative arrest in distal cells. In an *ex vivo* 3D skin equivalent model that has both dermal and epidermal layers recapitulating important features of human skin, senescent, but not non-senescent, melanocytes crucially led to reduced epidermal proliferation and epidermal atrophy (72). Furthermore, selective elimination of the senescent melanocytes or treatment with the mitochondrial targeted antioxidant MitoQ retarded this effect (72). Thus, a feed-forward positive loop enforces senescence and, through mitochondrial ROS generation, leads to skin atrophy—a hallmark of skin aging. In dermal fibroblasts, it has been demonstrated that age-related senescence is important in skin aging mediated by the depletion of stem cell pools within the tissue that maintain the epidermal dermal thickness (73). Mechanistically, mitochondria-derived ROS activated and upregulated the AP-1 transcription factor Jun B which then enforced senescence through the downregulation of IGF-1 and sustained P16 expression that reduced stem cell pools, leading to skin atrophy (73). Importantly, fibroblast-specific ablation of Jun B mitigated the senescence and retarded skin atrophy and collagen loss via stem cell pool restoration (73). Furthermore, a very recent study determined in a 3D skin equivalent model that senescence fibroblasts cross-talk with keratinocytes and decrease the stemness of basal keratinocytes. While not formally identified, it is likely that a soluble mediator(s) from the SASP exerts this effect (74).

Exposure to UVA or UVB radiation can accelerate skin aging (photoaging) and, in both culture and *in vivo* exposure, directly increased senescent cell accumulation (75). This appears to be related to the induction of ROS and DNA damage. Furthermore, the fragmented collagen can in itself promote further ROS generation (76), which could then promote further senescence. Interestingly, it was demonstrated that senescent dermal fibroblasts have accelerated accumulation in skin via SASP that suppresses their clearance by macrophages (77). It is known that aged dermal fibroblasts have significantly elevated levels of ECM degrading MMPs (78), and such MMPs are part of the SASP. Furthermore, elevated senescence markers are found in the epidermis of photoexposed skin across the decades (79). Are the extrinsic or intrinsic aging skin phenotypes linked by senescence? It is known that elevated ROS mediates senescence driving the bystander effect (17), which may induce a positive feedback loop enforcing senescence (80). This suggests that the selective removal of senescence or the SASP would retard the general features of skin aging such as atrophy and wrinkling. A note of caution must be taken here though as it was noted that senescent cells are important in normal wound healing response, and the depletion of these may impair wound healing and regeneration. Local versus systemic senolytic administration to ameliorate these effects is still unknown. Xeroderma pigmentosa is a rare genetic disorder that is caused by a base editing defect that results in UV-exposed skin defects due to dysfunctional base excision repair and also has elevated senescence in the skin (81). Recently, using the senolytic compound ABT-263 aged mice treated with ABT-263, reduction of senescent cells with an increase in collagen and epidermal thickness was demonstrated (82), suggesting that senotherapy can attenuate age-related skin reductions.

## Targeting of chronic senescent cells

As the evidence suggests, senescence is required to restore tissue integrity initially during wound repair, but lingering senescent cells can lead to a maladaptive repair response, so strategies to reduce senescent cells in the chronic situation would promote homeostasis. The duality of senescent cells is not altogether surprising and likely reflects a normal physiological response that, if not terminated, can be deleterious.

In preclinical models, elimination of senescent cells through senolytic therapy retarded many age-related diseases (6)—for example, age-related bone loss in mice is retarded with senolytic therapy (83). The first-described senolytics were the combination of dasatinib and quercetin that could retard age-related diseases such as cardiac function that declines with age and that extended the lifespan in progeroid mice (84). Dasatanib is a protein tyrosine kinase inhibitor, and quercetin is a plant flavonoid, and they are most effective in combination. The mechanism in cell elimination is targeting apoptotic pathways in the case of dasatinib. This targets pathways of Src kinases, and the flavonoid quercetin exerts its effects through the inhibition of the PI3K/Akt pathway, ultimately leading to the inhibition of anti-apoptotic pathways and thus apoptosis (85). Navitoclax is another identified senolytic that eliminates senescent cells primarily through targeting the Bcl-2 family of anti-apoptotic proteins including Bcl-2 and Bcl-xl (86) and has undergone phase II clinical trials for lymphoid cancers. Recently, it was shown that ectonucleotide pyrophosphatase/phosphodiesterase family member 5 (ENPP5) was a key factor in senescence in dermal fibroblasts, and knockdown of ENPP5 retarded the age-related thinning of collagen in mouse skin (87). Mechanistically, it is not clear how ENPP5 is driving senescence, but its function is to cleave NAD; so, it could be speculated that inhibition reduced NAD breakdown, leading to replenished NAD levels (88). Cardiac glycosides have also been demonstrated to be senolytics (89). **Table 1** shows the current senolytics.

**Table 1:** Current senolytic drugs.

Compound	Reference
Dasatinib	(84)
Quercetin	(84)
ABT-236 (Navitoclax)	(90)
ABT-737	(91)
Fisetin	(84, 92)
Geldanamycin	(93)
FOX04-DRI	(94)
17-DMAG	(93)
UBX0101	(95)
Panobinostat	(96)
Cardiac glycosides	(89)
3-Deazaadenosine	(97)
Canagliflozin	(98)
Senescence-specific CAR T cells	(99)

In the context of fibrosis, senescent cells have been found at the site of lung fibrosis, and senescent cell elimination attenuated fibrosis. Senescent cells have also been identified in kidney fibrosis and liver fibrosis. Furthermore, in animal models of kidney fibrosis, it was shown that elimination of senescent cells in the repair phase of wound healing by the senolytic ABT-263 (Navitoclax) has led to loss of senescent cells and reduction of fibrosis in the kidney (57). Furthermore, in models of cardiac ischemia–reperfusion injury, it was found that senescent cardiomyocytes led to increased ECM deposition and that elimination of these senescent cells with navitoclax led to much reduced cardiac fibrosis (100). Indeed p16 cardiomyocyte-specific KO mice have reduced fibrosis after reperfusion injury (101). Agents that have shown senolytic abilities include inhibitors of Heat Shock Protein 90 such as geldanamycin and 17-AAG (93), and these have interestingly been shown to retard elevated skin fibrosis (102), which could be through the selective elimination of senescent cells in the skin. In diabetic mouse models, impaired cardiac regeneration is common, and in these models, targeted elimination with the senolytics dasatanib and quercetin restored normal cardiac regeneration via the reduction of senescent cells and subsequent SASP release (103).

Indeed a phase 1 clinical trial of the senolytic cocktail dasatanib and quercetin in treating idiopathic pulmonary fibrosis demonstrated good safety with high tolerability, but larger randomized control trials are needed to determine the efficacy (104). There are over 30 clinical trials ongoing currently with senolytics in a variety of diseases. Furthermore, aside from senolytic agents that directly target senescent cell elimination through the modulation of apoptosis-inducing proteins (105), there is another class of compounds called senomorphs that modulate not the cells but the SASP. Therefore, senomorphs reduce the aberrant effects of the SASP as opposed to the senescent cell itself. Such senomorphs include the selective FDA-approved JAK1/2 inhibitor ruxolitinib (20, 106). Furthermore, blocking the mTOR pathway with rapamycin retarded the SASP from senescent cells through blocking IL-1 translation; therefore, rapamycin is a positive senomorphic drug (65). It also appears that the senomorphic blockade of the SASP by rapamycin was also mediated by blocking STAT3 activation (107). A highly novel approach to selectively eliminate senescent cells has been used using chimeric antigen receptor T cells (CAR T cells) targeting the uPAR receptor; this showed clinical benefit in an animal model of lung cancer (99). This may be a novel approach as a senolytic, although CAR T therapy is expensive and requires immunosuppression. **Table 2** demonstrates interventions that have targeted senescent cells *in vivo* in skin. **Table 3** demonstrates clinical trials using senolytics in diseases in which there is deleterious wound healing and fibrosis, but no clinical trial targeting the skin with serotherapeutics exists. Given that most data regarding senescence and wound healing are derived from animal models, its relevance to human skin must be evaluated.

**Table 2:** Interventions *in vivo* targeting senescent cells in skin.

Senescence targeting	Intervention	Reference
Yes	p163MR mice which is a mouse model in which genetic deletion of senescent cells can be performed. In acute wound healing, it was demonstrated that senescent cells are necessary for efficient wound healing.	(31)
Yes	Genetic KO of P16 and P21 double KO mouse to deplete senescent cells demonstrated retarded wound healing in the cutaneous wound model.	(31)
Yes	CCN1 knock in mouse in which the *Ccn1* genomic locus is replaced by an allele encoding DM, a CCN1 mutant with the α_6_β_1_-HSPG binding sites disrupted by alanine substitutions, thus rendering this inactive. This leads to reduced senescent cells and overactive cutaneous wound healing, ultimately leading to fibrosis. Mechanistically, CCN1 leads to fibrosis via induction of senescent via increased ROS and DNA damage.	(8)
Yes	Senolytic ABT-263/navitoclax was used *in vivo* in an animal model of skin fibrosis, the bleomycin model of skin fibrosis. ABT-263 led to reduced fibrosis in this skin model associated with less collagen and reduction of myofibroblasts.	(63)
Yes	ABT-263 and ABT-737 senolytics were used in aged hairless mice. These senolytics led to the selective removal of senescent cells which increased dermal collagen content, epidermal thickness, and keratinocyte proliferation, alongside the repression of inflammatory cytokines (e.g., IL-6) and matrix-degrading MMPs.	(82)

**Table 3:** Clinical trials in fibrotic disease with senolytics.

Disease	Intervention	Identification of study	Outcome
Systemic sclerosis pulmonary fibrosis	Dasatinib	NCT00764309	Completed. Treatment was associated with a good safety profile but no significant clinical efficacy (108)
IPF	Dasatinib and quercetin	NCT02874989	Completed. Good clinical safety profile, possible clinical benefit (109)
Non-alcoholic fatty liver disease with fibrosis	Dasatinib and quercetin	NCT05506488	Currently recruiting

## Conclusion

Senescent cells appear to play a dichotomous role in wound healing; on the one hand, they are a prerequisite for efficient healing through the SASP (31), but a chronic persistence within the wound leads to abnormal responses in either chronic non-healing wounds (42, 47) or fibrosis (63), with elevated levels of senescent cells seen in the skin fibrotic disease SSc and could be removed with senolytics (62). It is likely that factors, such as growth factors, underpin the regenerative role of the SASP (35), with PDGF-A being identified as important (31). It is possible that other SASP factors are key in the acute regenerative setting. Finally, a deeper understanding of the drivers of persistent senescence in chronic wounds is necessary so that upstream drivers can be exploited therapeutically and how they elicit their pro-senescent effects can be established. A study of acute versus chronic senescent cell removal in wound healing would greatly aid our understanding. Recently, apoptotic stress and the release of mitochondrial DNA have been demonstrated to drive senescence (110). This release via mitochondria pores of this DNA drives the SASP and senescence via activation of the Stimulator of Interferon Genes (STING) immune regulator (110). It could be that in aged non-healing wounds senescent cell burdens persist because of apoptotic stress leading to cytosolic DNA triggering the STING pathway. In the context of skin aging and senescence mediating the classic signs of aging such as epidermal thinning, direct irrefutable evidence of senescence governing this is currently lacking. Accumulating evidence indicates the presence of senescent cells with a prominent SASP in aged skin, but linking this to phenotypic alterations is lacking (69). The intimate interplay between senescent cells, their pro-inflammatory SASP, and interactions with immune cells in wound healing is obscure and requires further attention—for example, what is the relative contribution of each specific senescent cell type? Melanocytes are a key cell type in the skin, but relatively little research has focused on this cell type in relation to aging and senescence. Further research understanding the links between aging, senescence, and wound healing will illuminate pathways that can be targeted.
